# Proteomics analysis of methionine enkephalin upregulated macrophages against infection by the influenza-A virus

**DOI:** 10.1186/s12953-023-00205-w

**Published:** 2023-04-11

**Authors:** Wenrui Fu, Zifeng Xie, Mei Bai, Zhen Zhang, Yuanlong Zhao, Jing Tian

**Affiliations:** 1 Graduate College, Jinzhou Medical University, Jinzhou, 121000 China; 2 First Clinical Medical College, Jinzhou Medical University, Jinzhou, 121000 China; 3 Department of Microbiology, Jinzhou Center for Disease Control and Prevention, Jinzhou, 121000 China; 4 Department of Immunology, School of Basic Medical Sciences, Jinzhou Medical University, Jinzhou, 121000 China

**Keywords:** Macrophage, MENK, Proteomics analysis, Polarization, Phagosome

## Abstract

Macrophages have a vital role in phagocytosis and antiviral effect against invading influenza viruses. Previously, we found that methionine enkephalin (MENK) inhibited influenza virus infection by upregulating the “antiviral state” of macrophages. To investigate the immunoregulatory mechanism of action of MENK on macrophages, we employed proteomic analysis to identify differentially expressed proteins (DEPs) between macrophages infected with the influenza-A virus and cells infected with the influenza-A virus after pretreatment with MENK. A total of 215 DEPs were identified: 164 proteins had upregulated expression and 51 proteins had downregulated expression. Proteomics analysis showed that DEPs were highly enriched in “cytokine-cytokine receptor interaction”, “phagosome”, and “complement and coagulation cascades pathway”. Proteomics analysis revealed that MENK could be an immune modulator or prophylactic for the prevention and treatment of influenza. MENK promoted the polarization of M1 macrophages, activated inflammatory responses, and enhanced phagocytosis and killing function by upregulating opsonizing receptors.

## Introduction

There are four types of influenza viruses: A, B, C and D. The influenza-A virus is a contagious pathogen responsible for severe respiratory disease in humans, and its prevalence continues to pose a major threat to public health worldwide [1, 2]. Vaccination is currently the best method to protect against morbidity and mortality from inflfluenza infection. However, due to insufficient vaccination coverage, vaccine shortage and mismatch with the circulating virus strains, vaccination failed to provide complete protection, particularly in high risk populations [3, 4]. Antivirals represent an important prophylactic and therapeutic measure to reduce influenza-associated morbidity and mortality. Immunomodulatory interventions illustrate the potential utility of developing next-generation antivirals against influenza [5]. Cells of the innate immune system and adaptive immune system are the main effector cells in the fight against and elimination of influenza viruses. The former recognize antigens in a non-specific manner, and phagocytose and eliminate cells infected by different influenza strains. With regard to the adaptive immune system, T-cell responses mainly target the conserved viral-epitope proteins of NP, PA, PB1, and PB2 of the influenza virus, and can respond to heterogeneous influenza infections.

A dendritic cell (DC)-targeting vaccine has received global attention recently. It directed hemagglutinin and the chemokine receptor Xcr1 + of the influenza virus to DCs to induce an immune response and confers protection against the influenza virus [6, 7]. Macrophages are the key cells for the surveillance, defense, and homeostasis of the immune system. Macrophages activate and engulf influenza virus-infected cells to limit the spread of the influenza virus. Moreover, macrophages “present” viral proteins to T cells and initiate a T cell mediated cellular immune response, which is crucial for eliminating viruses. Studies have demonstrated the importance of alveolar macrophages in establishing a broad spectrum “antiviral state” [8]. Two specific rAd-Sn4D-Fc and rAd-SRCR59-Fc of alveolar macrophages can be developed further as therapeutic vectors to facilitate eradication of the porcine reproductive and respiratory syndrome virus [9]. M2e5x immune sera-stimulated macrophages can secrete cytokines through fragment crystallizable (Fc) receptors and cross-protect against the influenza-A virus subtypes H1N1, H3N2, and H5N1, indicating a possible mechanism of influenza protection [10]. Therefore, macrophages, as innate immune cells, can also be the target for the development of influenza prophylactics or vaccines.

Methionine enkephalin (MENK) is an endogenous neuropeptide, composed of Tyr-Gly-Gly-Phe-Met, derived from the prohormones and proenkephalins produced by the adrenal glands, with neuroendocrine regulatory activity, strong immunoregulatory activity and non-cytotoxic [11, 12]. MENK is present in the blood and nervous system in low concentrations and has been synthesized artificially now. Researchers have found that MENK positively regulates the functions of eosinophils, basophils, neutrophils, natural killer cells, DCs, macrophages, and T cells [13–15]. Our previous studies have proven that MENK in prophylactic administration, up-regulated the status of macrophages before infection. Once the influenza virus invaded, induced macrophages rapidly exerted immunobiological effects to recognize, phagocytose virus and release inflammatory cytokines [16]. Experiments of prophylactic MENK inhibited influenza virus infection in vivo also supported this view [17].

Our laboratory data have illustrated that MENK could be working as a non-specific prophylactic to be used for influenza epidemic. In order to further elucidate the molecular mechanism of MENK as immune modulator or prophylactic on macrophages, we employed proteomics analysis and bioinformatics analysis to identify differentially expressed proteins (DEPs) between macrophages infected with the influenza-A virus and cells infected with the influenza-A virus with MENK pretreatment (pre-MENK).

## Materials and methods

### Viruses and cells

The influenza-A virus, A/PR/8/34 (H1N1), was kindly provided by the China Center for Disease Control and Prevention (Beijing, China). A murine macrophage cell line (RAW264.7) was purchased from the Cell Resource of Chinese Academy of Sciences (Shanghai, China).

### Cell infection and treatment with MENK

RAW264.7 cells were divided into two groups: influenza A virus-infected control (H1N1-C) and pre-treatment with MENK (pre-MENK). H1N1-C group cells were inoculated with H1N1 at a median tissue culture infectious dose (TCID_50_) of 100 for 1 h and washed with phosphate-buffered saline (PBS). Then, they were cultured in Dulbecco’s modified Eagle’s medium (DMEM; Gibco, Grand Island, NY, USA) containing 1% bovine serum albumin (Gibco), penicillin (100 U/mL), streptomycin (100 μg/mL), HEPES (25 mM; Gibco), and tosyl phenylalanyl chloromethyl ketone-trypsin (2 μg/mL; MilliporeSigma, Burlington, MA, USA) for 72 h. Pre-MENK group cells were treated with MENK (≥ 99% purity; 10 mg/mL; American Peptide, Sunnyvale, CA, USA) in DMEM containing 10% fetal bovine serum (Gibco) for 48 h. Then, they were washed with PBS, inoculated with H1N1 (100 TCID_50_) for 1 h, washed with PBS, and allowed to culture for 72 h. Cells were collected 72 h after virus infection and stored at − 80℃.

### Extraction and digestion of proteins

Samples were thawed from − 80℃ to room temperature. Four-times volume of lysis buffer containing urea (8 M) and a 1% protease inhibitor cocktail was added. Cells were lysed by sonication. The remaining debris was removed by centrifugation at 12,000 × *g* for 10 min at 4℃. Finally, we collected the supernatant and measured the protein concentration with a bicinchoninic-acid kit.

For digestion, the protein solution was reduced with dithiothreitol (5 mM) for 30 min at 56 °C and alkylated with iodoacetamide (11 mM) for 15 min at room temperature in the dark. Then, the protein sample was diluted by addition of tetraethylammonium bromide (100 mM) to facilitate a urea concentration < 2 M. Finally, trypsin was added at a mass ratio of trypsin:protein of 1:50 for the first digestion overnight and a mass ratio of trypsin:protein of 1:100 for a second 4 h-digestion.

### Liquid chromatography-tandem mass spectrometry (LC–MS/MS)

Tryptic peptides were dissolved in solvent A (0.1% formic acid, 2% acetonitrile in water) and loaded directly onto a homemade reversed-phase analytical column (length = 25 cm, 100 μm i.d.). Peptides were separated with a gradient from 4 to 23% solvent B (0.1% formic acid in 90% acetonitrile) over 62 min, 23% to 35% in 20 min, climbing to 80% in 4 min, and then holding at 80% for the final 4 min, all at a constant flow rate of 500 nL/min on an EASY-nLC 1200 UPLC system (Thermo Fisher Scientific, Waltham, MA, USA).

Separated peptides were analyzed an a Q Exactive™ HF-X mass spectrometer (Thermo Fisher Scientific) with a nano-electrospray ion source. The electrospray voltage applied was 2.1 kV. The full MS scan resolution was set to 120,000 for a scan range of 400–1500 m*/z*. The 10 most-abundant precursors were selected for further MS/MS analyses with a dynamic exclusion of 30 s. Higher-energy C-trap dissociation was undertaken at a normalized collision energy of 28%. Fragments were detected in the Orbitrap at a resolution of 15,000. The automatic gain control target was set at 5E4, with an intensity threshold of 2.5E5 and a maximum injection time of 40 ms.

### Protein quantifification and criteria for protein identifification

The resulting MS/MS data were processed using MaxQuant 1.6.15.0 (www.maxquant.org/). Tandem mass spectra were searched against the Mus_musculus_10090_SP_20201214. fasta database concatenated with reverse decoy database. Trypsin/P was specified as the cleavage enzyme, with up to two missing cleavages being allowed. The mass tolerance for precursor ions was set as 20 ppm in the first search and 5 ppm in the main search. The mass tolerance for fragment ions was set as 0.02 Da. Carbamidomethyl on Cys was specified as a fixed modification. Acetylation on protein N-terminal, oxidation on Met and deamidation (NQ) were specified as variable modifications. The false discovery rate was adjusted to < 1%. The minimum score for peptides was > 40. We changed the label-free quantitation (LFQ) intensity (*I*) level of the proteins in different samples through centralization to obtain the relative quantitative value (*R*) of the protein. The calculation formula is *R*_ij_ = *I*_ij_/Mean(*I*_j_),where i denotes the sample and j denotes the protein. H1N1-C group (Group A) was set as the control sample, and pre-MENK group (Group B) was treated sample. We used three standard biological replicates to test whether the quantitative results were consistent with statistical consistency. Pearson's correlation coefficient (PCC), principal component analysis (PCA) and relative standard deviation (RSD) were used to evaluate repeatability.

### Bioinformatics analysis

Fold change > 1.50 or < 0.67 and *P* < 0.05 were set as the significant thresholds for DEPs. Based on terms set in the Gene Ontology (GO) database (http://geneontology.org/), DEPs were annotated into three categories: biological process, cellular component, and molecular function. Analyses of enrichment of signaling pathways were undertaken using the Kyoto Encyclopedia of Genes and Genomes (KEGG) database (www.genome.jp/).

### Molecular docking

The related protein structures were downloaded from PDB, and MENK 2D structures were downloaded from Pubchem, and it was transformed from SDF formal to MOL2 using OpenBabelGUI. Subsequently, the receptors were dehydration using PyMOL software, with removing the other useless atoms. Next, molecular docking was performed in AutoDock to complete the docking and analysis. Then the docking results were exported in the PDBQT format. PyMOL software was used to calculate the length of hydrogen bonds and exported the ligand and the overall pictures.

## Results

### Quantification of proteins

**Fig. 1:**
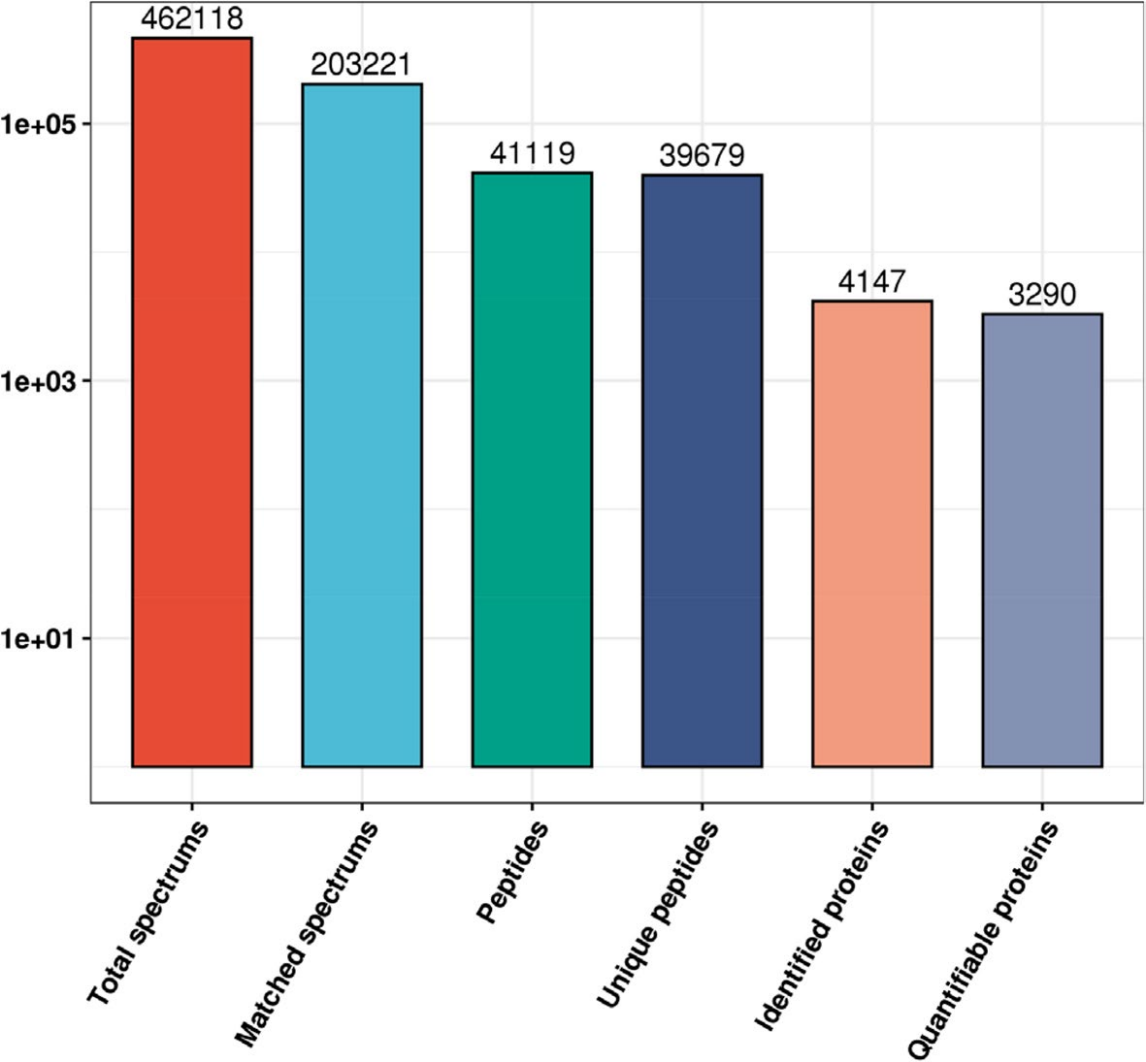
Overview of the mass spectrometry results. Bar plot summarizing the detected peptides and proteins in H1N1-C group and pre-MENK group

In total, 462,118 spectrograms were obtained by MS. The number of available effective spectrograms was 203,221, and 44.0% of them were utilized. A total of 41,119 peptides were identified by spectrogram analysis, among which 39,679 were specific peptide segments. A total of 4,147 proteins were identified, of which 3,290 were quantifiable (Fig. 1).

### Quality control of data

**Fig. 2:**
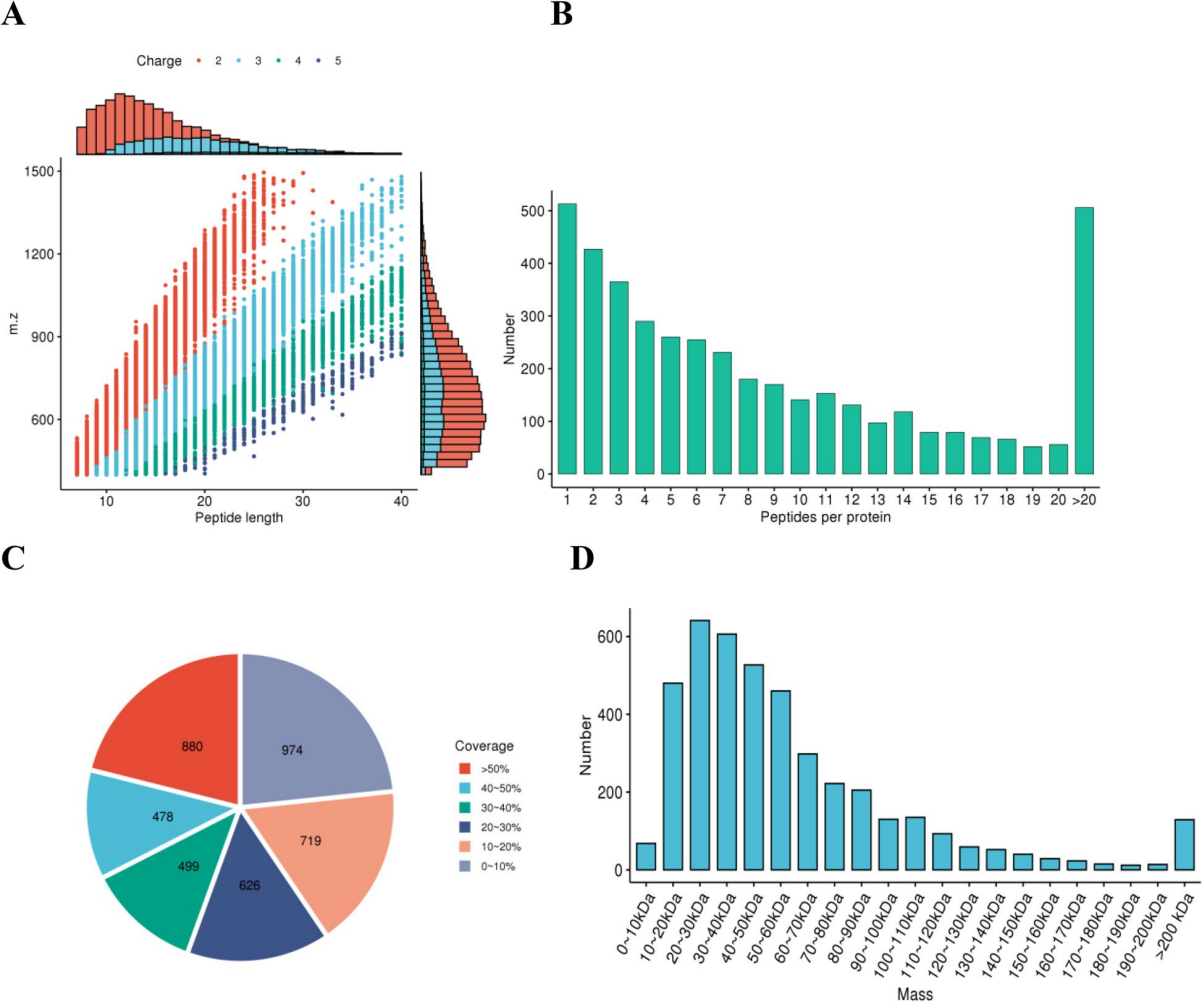
Peptide distribution. **A** Peptide length distribution. **B** Peptide number distribution. **C** Protein coverage distribution. **D** Protein molecular weight distribution

Quality control data showed most peptides were distributed in the range of 7–20 amino acids, the length distribution of peptides identified by mass spectrometry met the quality control requirements (Fig. 2A). Most proteins correspond to more than two peptides. In quantification, a protein corresponding to multiple specific peptides was conducive to increase the accuracy and credibility of the quantitative results (Fig. 2B). Most proteins had coverage below 30%. In the shotgun strategy of mass spectrometry, the mass spectrometry preferentially scanned peptides with higher abundances. Therefore, there was a positive correlation between protein coverage and abundance in the sample (Fig. 2C). The molecular weight of the identified proteins was uniformly distributed in different stages (Fig. 2D). The quality of the results met an acceptable standard based on the peptide length distribution, number distribution, coverage distribution, and molecular weight distribution.

### DEP analyses

**Fig. 3:**
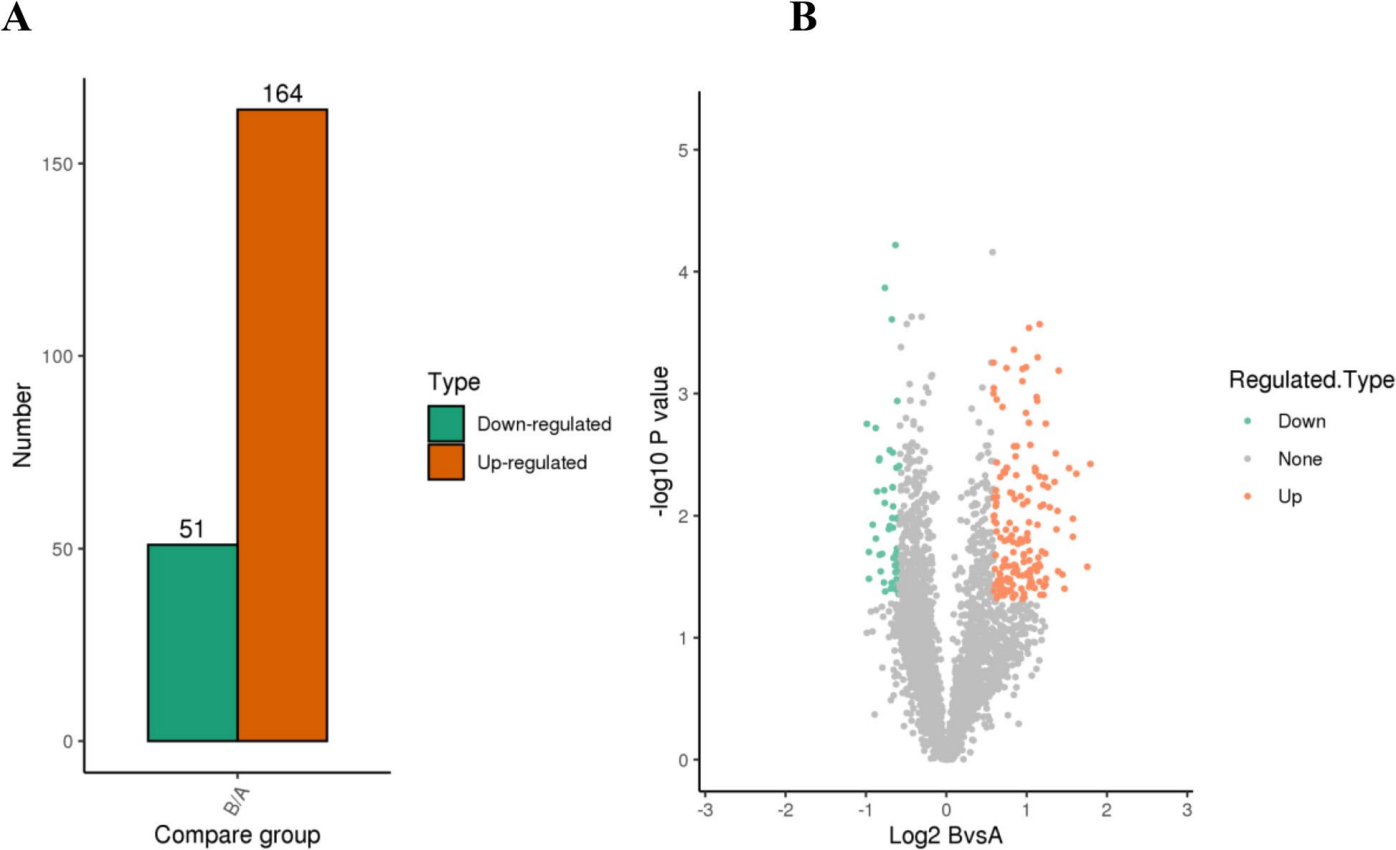
Identification of DEPs between H1N1-C group and pre-MENK group. **A** The total number of upregulated and downregulated DEPs. **B** Volcano plot of the identified DEPs between H1N1-C group and pre-MENK group. The red dots denote upregulated DEPs, the green dots denote downregulated DEPs, and the gray dots denote unchanged proteins

Compared with the H1N1-C group, 215 DEPs were identified in the pre-MENK group: 164 proteins had upregulated expression and 51 proteins had downregulated expression with fold change over 1.5 fold (Fig. 3).

### Enrichment analyses of DEPs

**Fig. 4:**
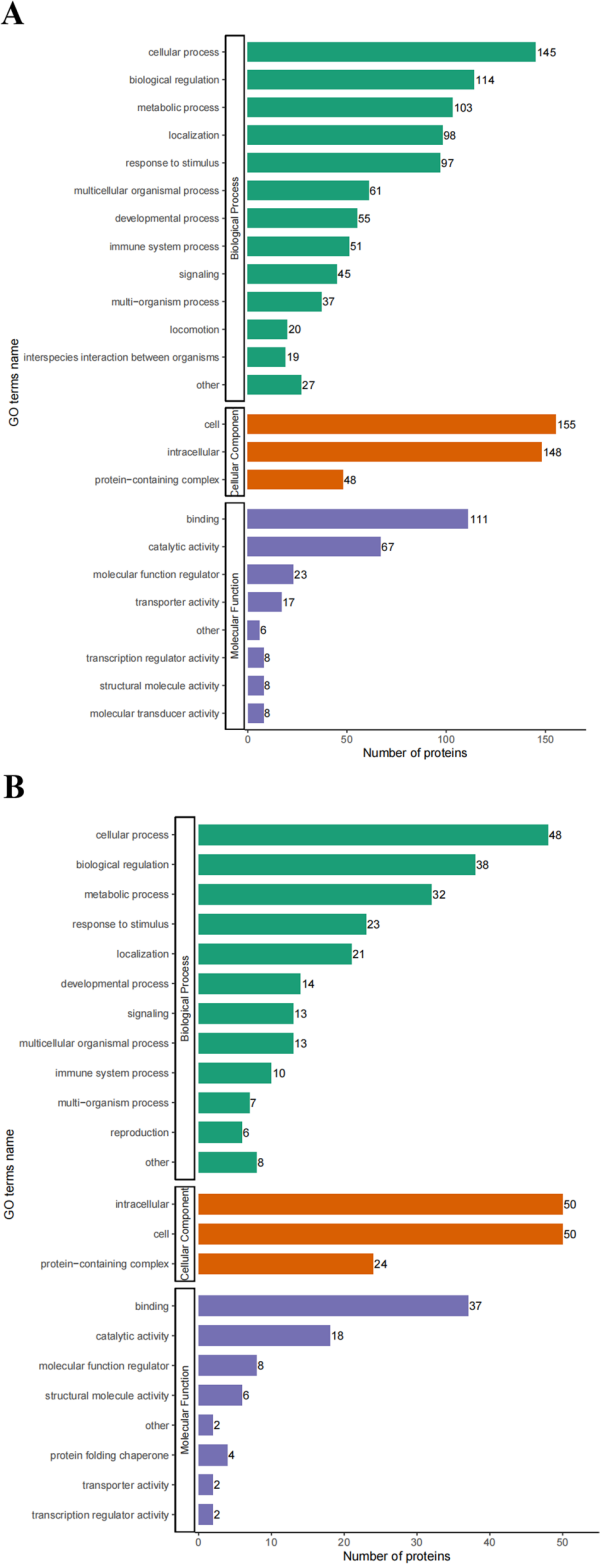
GO analysis for the identified DEPs. The DEPs were annotated into three categories based on GO terms, including biological processes, cellular components, and molecular functions. **A** GO enrichment analysis of the upregulated DEPs. **B** GO enrichment analysis of the downregulated DEPs

**Fig. 5:**
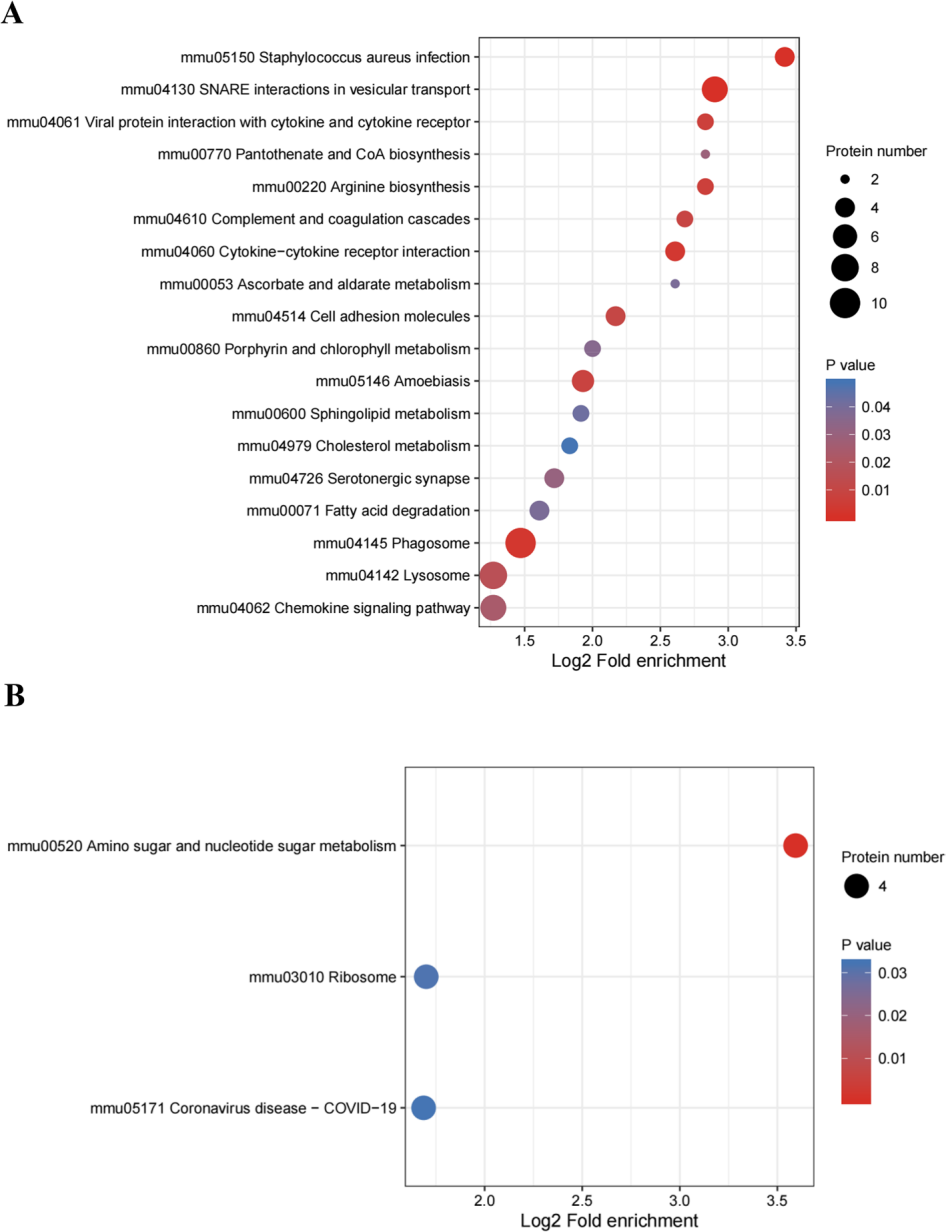
KEGG pathway enrichment analysis of DEPs. Bubble diagrams displaying KEGG pathways for signifificantly enriched upregulated. **A** and downregulated. **B** DEPs. The Y axis showed the IDs and names of enriched pathways, and the X axis showed the converted log2 fold enrichment. The size of the bubbles described the number of DEPs in pathway

**Fig. 6:**
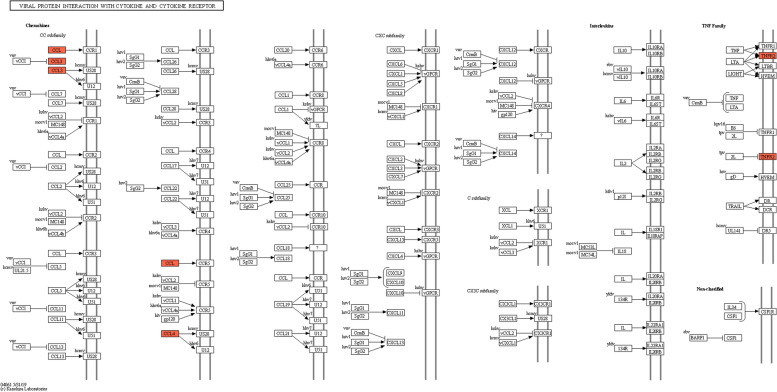
KEGG enrichment pathway. Viral protein interaction with cytokine and cytokine receptor (mmu04061). The red color represents the up-regulated protein

To ascertain if DEPs had significant enrichment in specific functional categories, we conducted functional-enrichment analysis of DEPs based upon GO and KEGG databases. Notably, the DEPs were significantly enriched for several relevant GO terms, including cellular process, response to stimulus and binding (Fig. 4). The enriched KEGG pathways were shown in Fig. 5. Among them, several macrophage polarization-related pathways were enriched, such as chemokine signaling pathway (mmu04062), cytokine-cytokine receptor interaction (mmu04060) and viral protein interaction with cytokine and cytokine receptor (mmu04061). The main pathway of inducing macrophage polarization was modulated by the interaction with cytokine and cytokine receptor, including CCL3, CCL4 and TNFR2 (Fig. 6).

**Fig. 7:**
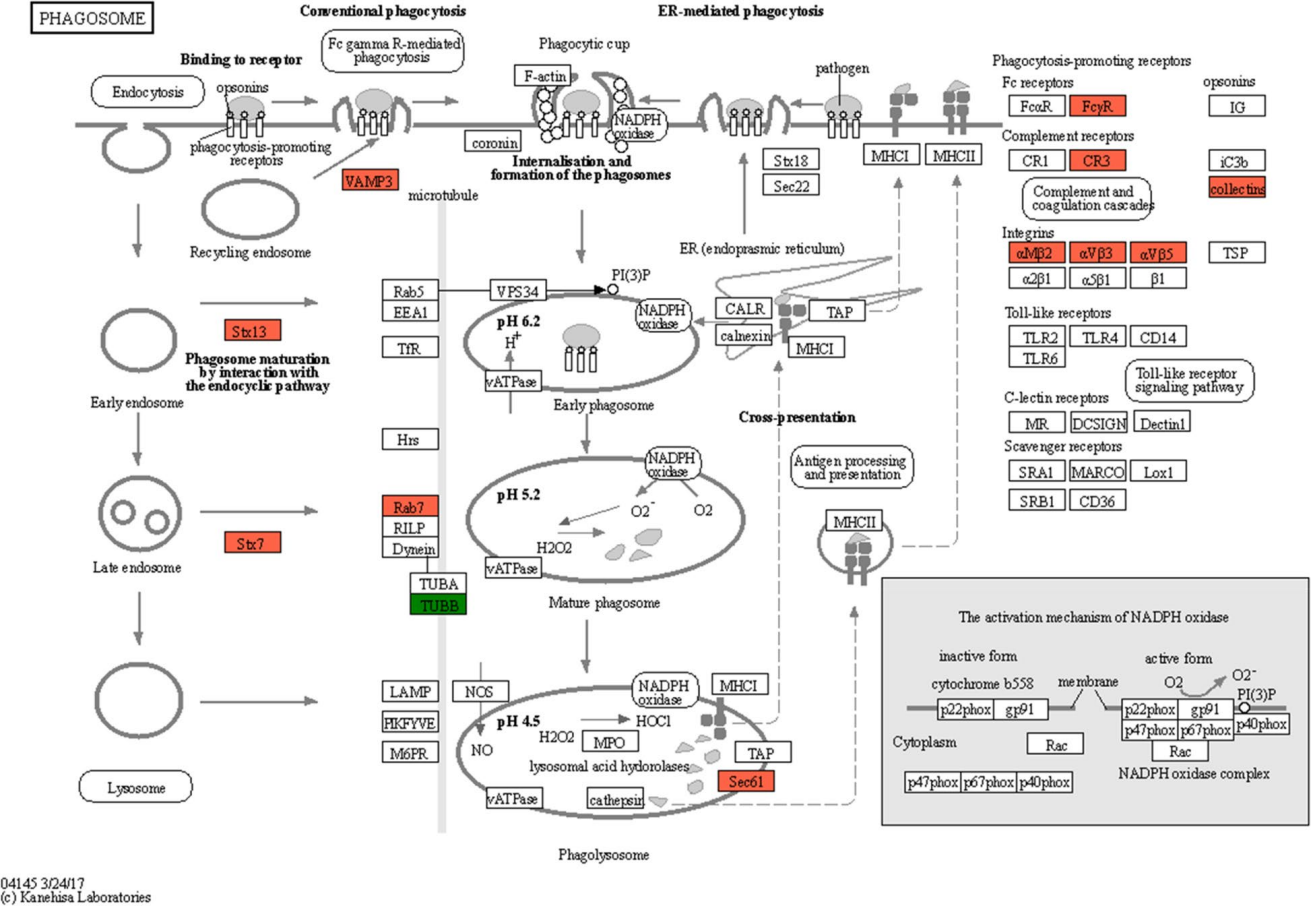
KEGG enrichment pathway. Phagosome (mmu04145). The red color represents the up-regulated protein, green color represents the down-regulated protein

Furthermore, receptor mediated endocytosis and phagosome formation-related pathways were phagosome (mmu04145) and complement and coagulation cascades (mmu04610), both of these pathways indicated that complement receptors and Fc receptors played a crucial role in receptor-mediated endocytosis. In Fc receptor-mediated endocytosis, downstream STX 13, STX7, and Rab7 were involved, while αvβ5 and αvβ3 were involved in complement-mediated endocytosis (Fig. 7).

The enrichment analyses suggested that pre-MENK regulated macrophages by macrophage polarization and receptor-mediated endocytosis.

### Hierarchical clustering analysis

**Fig. 8:**
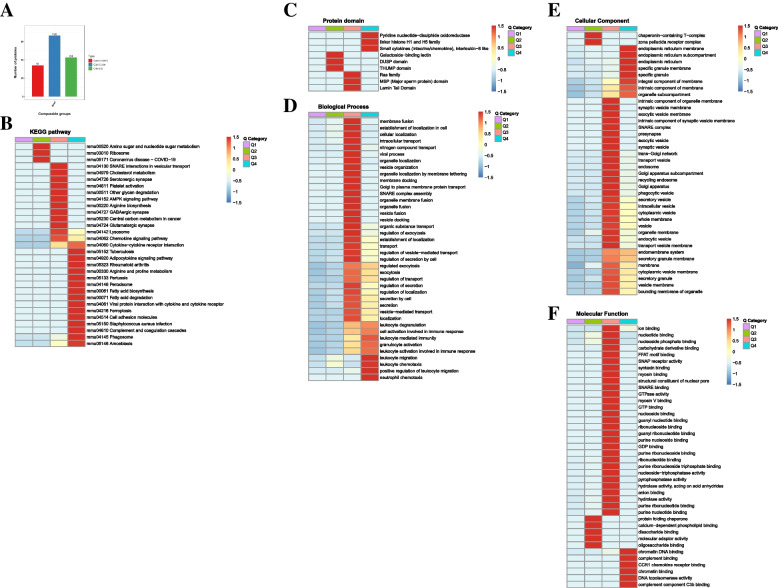
Hierarchical cluster analysis for the DEPs. **A** The significant thresholds for the DEPs were Fold change > 1.50 or < 0.67 and *P* < 0.05. The DEPs were divided into four groups from Q1 to Q4 according to the degree of FC. **B** The Q categories for KEGG pathways. **C** The Q categories for protein domain. **D** The Q categories for biological process. **E** The Q categories for cellular component. **F** The Q categories for molecular function. The red color indicates stronger enrichment. The blue color indicates weaker enrichment

According to the degree of fold change, DEPs were divided into four groups: “severely downregulated” (Q1, FC ≤ 0.5), “mildly downregulated” (Q2, 0.5 < FC ≤ 0.667), “mildly upregulated” (Q3, 1.5 < F ≤ 2.0), and “severely upregulated” (Q4, FC > 2.0) (Fig. 8A).

DEPs with severely upregulated expression were enriched for the “phagosome, complement and coagulation cascades”, “viral protein interaction with cytokine and cytokine receptor”, “cytokine–cytokine receptor interaction”, and “chemokine signaling pathway” (Fig. 8B), which were highly related to inflammatory cytokines, cytokine receptor and chemokines released by macrophages infected with influenza virus. In the clustering analysis for the protein domain, some DEPs with severely upregulated expression were enriched for “pyridine nucleotide-disulfide oxidoreductase”, and “small cytokines (integrin/chemokine), interleukin-8 like” (Fig. 8C). In the clustering analysis using the GO database, DEPs with severely upregulated expression of biological processes were enriched for “neutrophil chemotaxis”, “positive regulation of leukocyte migration”, “leukocyte chemotaxis”, and “leukocyte migration” (Fig. 8D). The clustering analysis of protein domains and biological process illustrated that chemokines induced the migration and activation of leukocyts, which may attribute to the mechanism of MENK up-regulating the antiviral function of macrophages. The main cellular components to be enriched were “endoplasmic reticulum” and “specific granule membrane” (Fig. 8E). The main molecular functions to be enriched were “complement component C3b binding”, “CCR1 chemokine receptor binding”, and “complement binding” (Fig. 8F), which indicated that complements were effective molecules for MENK to regulate the function of macrophages. We synthesized the hierarchical clustering analysis, and found that cytokines, cytokine receptors and chemokines, as well as complement were the main means of MENK to promote macrophage immune response, which were consistent with the results of enrichment analyses of DEPs.

### Analyses of protein-interaction networks

**Fig. 9:**
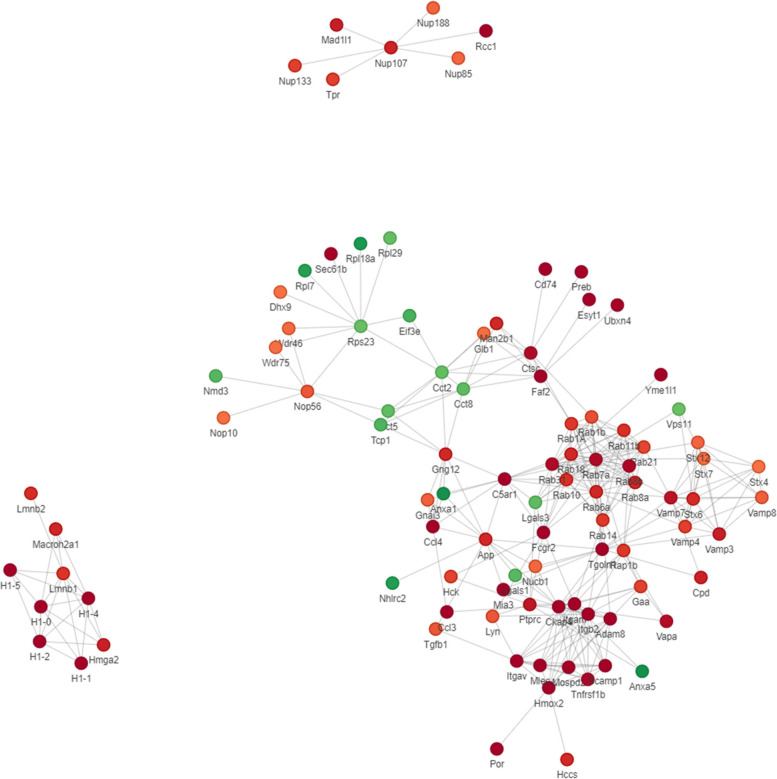
PPI network of the DEPs. Circles represent differential proteins, and different colors represent the differential expression of proteins. The darker the color, the larger the fold of difference

According to confidence score > 0.7 (high confidence), the differential protein interactions were extracted from 215 differential proteins. Visualization of the differential protein interaction network was performed using the R package "networkD3" tool. In order to clearly demonstrate the protein–protein interaction, we screened proteins with the closest interaction relationship and mapped the protein interaction network. In Fig. 9, circles represent differential proteins, and green color represented downregulated proteins, red color represented upregulated proteins. PPI showed close protein interactions with CCL3, CCL4, and TNFR2 in the cytokine-cytokine receptor pathway, and with CR3 (Itgam), upstream and downstream relation among FcγR, Rab7, STX13 and STX7 in the receptor-mediated endocytosis pathway. Protein interaction was also found among CCL3, TNFR2, FcγR and CR3. These proteins were also shown in the KEGG pathway map of viral protein interaction with cytokine and cytokine receptor (mmu04061) and phagosome (mmu04145). The visualization results of PPI are further illustrated that the targets of MENK on macrophages were related to chemokines and receptors with the function of mediating phagocytosis.

### Molecular docking

**Fig. 10:**
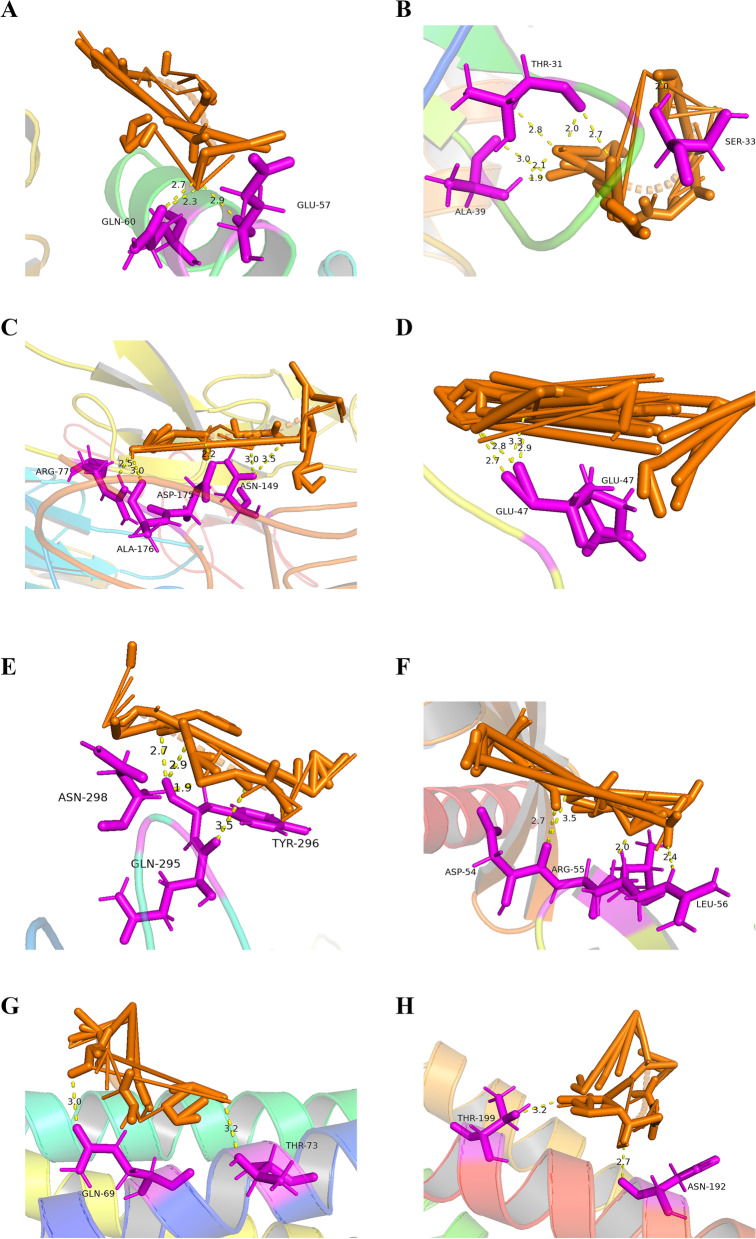
Molecular docking simulation diagram. **A** MENK-CCL3. **B** MENK-CCL4. **C** MENK-TNFR2. **D** MENK-CR3. **E** MENK-FCγR. **F** MENK-Rab7. **G** MENK-STX13. **H** MENK-STX7. The MENK is orange stick models and the protein molecules at the docking site are represented as violet stick models. The connected hydrogen bonds are indicated by yellow dotted lines

Based on the data of PPI, and combined with KEGG pathway(mmu04061 and 04145), we evaluated the interaction between MENK and 8 small-molecule core proteins. CCL3, CCL4, TNFR2, CR3, FcγR, Rab7, STX13 and STX7 proteins crystal structures were downloaded from the PDB database, improved by PyMOL, and molecular docking of the target proteins to the related ingredients was performed in AutoDock. Affifinity was the score used by the software to calculate the binding ability. It is generally believed that affinity < -7 kcal/mol indicated stronger binding activity, -7 < affinity ≤ -4 kcal/mol indicated moderate binding activity, affinit > -4 kcal/mol indicated week binding activity [18]. After calculation, it was found that the binding activity of MENK with core proteins were CCL4(-8.88), CCL3(-5.02), TNFR2(-5.86), CR3(-6.7), FcγR(-4.99), Rab7(-6.83), STX13(-4.77) and STX7(-5.06), respectively. Finally, PyMOL software was used to calculate the length of hydrogen bonds, improve and export the pictures. CCL3, CCL4, TNFR2, CR3, FcγR, Rab7, STX13 and STX7 were successfully docking with MENK (Fig. 10 A-H). In addition, we found the hydrogen bonds formed between molecules and represented by yellow dotted lines in the diagrams. These results indicated that the target of MENK fuction may be CCL3, CCL4, TNFR2, CR3, FcγR, Rab7, STX13 and STX7.

## Discussion

Macrophages play a leading part in pathogen recognition, and protect host defense through inflammation initiation and clearance of pathogens in the airways. Macrophages can be subdivided into classically activated (M1) and alternatively activated (M2) phenotypes according to their functional characteristics [19, 20]. In the local microenvironment, the pathogen and their products binding to macrophage surface receptor to generate signals or under the stimulation of cytokines, macrophage polarizes into M1 macrophages, which have strong phagocytosis and killing ability [21]. M1 macrophages are rich in lysosomal particles, which can directly kill and eliminate pathogens through nitrous oxide and release lysosomal enzymes, or secrete chemokines and pro-inflammatory cytokines mediate inflammatory response. Kapellos and colleagues reported that M1 macrophages secrete proinflammatory cytokines and chemokines, such as tumor necrosis factor (TNF)-α, interleukin (IL)-6, IL-12, chemokine (C–C motif) ligand (CCL)4, and C-X-C motif chemokine ligand (CXCL)10, and induce phagocytosis and oxidative-dependent killing mechanisms [22].

Consistent with those findings, we revealed significant enrichment of leukocyte chemotaxis, as well as the migration and activation of cells involved in the immune response, whereas the small cytokine (integrin/chemokine)/IL-8-like domain was enriched markedly according to clustering analysis of the protein domain. Furthermore, several processes related to the cytokine response, such as “viral protein interaction with cytokine and cytokine receptor (mmu04061)” and “chemokine signaling pathway” (mmu04062), were found to be enriched. MENK in prophylactic administration upregulated expression of the cytokines CCL3, CCL3L1, CCL4, CCL4L1, CCL4L2, and tumor necrosis factor receptor type (TNFR)2. CCL3, CCL3L1, CCL4, and CCL4L1 are family members related to chemokines [23]. CCL3 encodes macrophage inflammatory protein-1α, which plays a part in inflammation by binding to CCR receptors, as well as influencing the protein kinase B signaling pathway and CCR5 pathway in macrophages [24]. CCL4 (also known as macrophage inflammatory protein-1β) has diverse effects on various types of immune and nonimmune cells by interacting with CCR5, in collaboration with CCL3 and CCL5. Several lines of evidence indicate that CCL4 can enhance tumor immunity by recruiting cytolytic lymphocytes and macrophages with phagocytic ability [25]. Upon infection with influenza viruses, CCR5 and its cognate chemokines CCL3, CCL4, and CCL5 are induced rapidly, which contributes to leukocyte recruitment into the airways and a consequent efficacious antiviral response [26]. TNF-α is a pleiotropic cytokine produced mainly by macrophages, T cells, and neutrophils. It is crucial for inflammatory and immune responses in infectious diseases [27, 28]. TNFR1 is considered to be a “death receptor”and promotes the inflammatory response, cell survival, or death pathways. TNFR2 is associated with the promotion of cell survival and tissue homeostasis [29]. Our proteomics-analysis results revealed the TNFR2 level in macrophages to be enhanced. TNF-TNFR2 signaling helps macrophages to resist apoptosis and maintain homeostasis. Brenner and coworkers demonstrated TNF-TNFR signaling in protective immunity against *Orientia tsutsugamushi* infection [30]. PPI showed close protein interactions with CCL3, CCL4, and TNFR2 in the cytokine-cytokine receptor pathway. Therefore, we speculate that MENK in prophylactic administration enhanced the antiviral status of macrophages before virus infection by inducing M1 polarization and upregulating expression of the chemokines secreted by macrophages, which promote inflammatory reactions and leukocyte chemotaxis. Furthermore, MENK promotes TNFR expression on the surface and inhibits the apoptosis of macrophages, thereby enabling macrophages to have a stable antiviral function.

Cytokines secreted by M1 macrophages have a regulatory role in expression of the receptor FcγRs. In general, proinflammatory cytokines increase the level of activation of FcγRs [31]. Furthermore, FcγRs induces activation of proinflammatory macrophages (M1). The blockade of Fc-receptors for FcγRs of immunoglobulin (Ig)G reduces the production of proinflammatory cytokines, which suggests a potential role of FcγRs for the “reprogramming” of M2 macrophages [32]. Our proteomics-analysis results also confirmed this claim. PPI demonstrated the interaction between CCL3, TNFR2, FcγR, and CR3, indicating the synergistic interaction between macrophage secreted cytokines and receptors mediating endocytosis by prophylactic administration of MENK. Overall, the cytokines secreted by M1 macrophages and FcγRs are promoted and activated mutually. Studies have shown that FcγRs (as the best characterized phagocytic receptor on macrophages) can recognize the Fc segment of IgG and trigger a wide range of downstream effector functions. The crosslinking of FcγR on phagocytes induces IgG to modulate the phagocytosis of virus particles and infected cells. In this process, phagocytic virus particles/cells are degraded by acidification of phagosomes and digestion of lysosomal enzymes, release proinflammatory mediators, and produce cytokines [33, 34]. The leukocytic integrin αMβ2 (cluster of differentiation (CD11b/CD18), also known as CR3, is the main receptor of the complement fragment iC3b. It adheres to the surface of pathogens and promotes the phagocytosis of pathogens by binding to the I domain of CR3 on host immune cells [35, 36]. The ligation of CR3 triggers activation of Syk in host cells via an immunoreceptor tyrosine-based activation-like motif, which subsequently causes the activation of downstream pathways, thereby resulting in phagocytic effects [37]. The complement system represents the first line of defense because it opsonizes and subsequently lyses pathogens, inducing the secretion of proinflammatory cytokines and maintenance of homeostasis. Recently, complement was found to augment and modulate adaptive immunity significantly, in particular against viruses [38]. αvβ5 and αvβ3, which belong to the integrin family with CR3, also participate in the phagosome pathway (mmu04145). The integrin αvβ5 acts as a receptor for the phagocytic uptake of apoptotic cells and can activate the molecules involved in tissue homeostasis. The integrin αvβ3 has been shown to have an essential role in the metastasis, angiogenesis, hemostasis, and phagocytosis of tumor cells [39]. We can conclude that Fc receptors and complement receptors are involved in promoting macrophage phagocytosis of pathogens. In particular, Fc receptors can further induce M1 polarization of macrophages. Therefore, MENK upregulated these surface molecules and then promoted phagocytosis of macrophages and M1 polarization.

KEGG enrichment pathway (mmu04145) showed that FcγR, CR3, Rab7, STX13 and STX7 related to the processing of endosomes and phagosome formation was upregulated, and there were upstream and downstream relation among these molecules displaying by the PPI. “Phagosome formation” refers to an internalization process in which larger particles (e.g., bacteria and dead/dying cells) are engulfed and processed within a membrane-bound vesicle [40]. Vesicle-associated membrane protein-3 has an important role in the recycled endosomes that form an extensive and complex network of sub-compartmentalized vesicular and tubular elements which connect with the cell surface and other endosomes in macrophages [41]. Nascent phagosomes must undergo a series of fusion and fission reactions to acquire the microbicidal properties required for the innate immune response. Syntaxin (STX)13 ensures phagosome maturation by interacting with the endocyclic pathway. STX7 participates in the trafficking and fusion of endosomes. Rab7 is a regulator of endosome maturation [42]. Rab7 is involved in the biogenesis of phagosomes, which are crucial for microbial killing and antigen presentation [43]. Active Rab7 on the phagosome membrane is associated with Rab7-interacting lysosomal protein, which bridges phagosomes with a microtubule-associated dynein–dynactin motor complex [44]. The increased expression of proteins related to the formation of endosomes and phagosomes suggests that pre-MENK administration promotes the intake, processing, degradation, and elimination of antigens. In particular, MENK enhances antigen presentation by upregulating expression of Rab7, which is crucial for macrophage-activated T cells to mediate adaptive immune responses against influenza viruses.

## Conclusions

Our proteomics-analysis data on macrophages demonstrate that prophylactic administration of MENK may play an important part in upregulating antiviral state of macrophage before influenza viruses infection. In addition to our previous study reporting that MENK can promote macrophage polarization and increase cytokine secretion, our proteomic results suggest that macrophage receptor-mediated endocytosis and phagosome formation may be regulated by pre-MENK administration. Besides, MENK promotes the adaptive immune response indirectly by improving the antigen presentation of macrophages. Therefore, we speculate that MENK acts on innate immune cells with a non-specific, antigen-eliminating function. Prophylactic MENK should improve the antiviral status of macrophages in advance, which is conducive to the clearance process of all strains of influenza viruses. Hence, MENK could be used as an immune modulator or non-specific prophylactic for the prevention and treatment of influenza.

## Data Availability

The datasets presented in this study can be found in the article/supplementary material.
